# The Thorny Issue of Atypical Anorexia Nervosa: Clinicians' Perspectives on How It Should Be Defined

**DOI:** 10.1002/erv.3152

**Published:** 2024-11-18

**Authors:** Jessica Beard, Glenn Waller

**Affiliations:** ^1^ Department of Psychology University of Sheffield Sheffield UK

**Keywords:** classification, EDNOS, qualitative research

## Abstract

**Objective:**

Atypical Anorexia Nervosa (AAN) is an ill‐defined diagnosis. Little is known about how eating disorder clinicians perceive the utility of the diagnosis, and what changes they believe would add to that utility. This qualitative study aimed to explore clinicians' perspectives on refining the DSM‐5 AAN diagnosis.

**Methods:**

Content analysis of text was used to categorise 47 responses to the questions: “What changes are required to the DSM‐5 definition of AAN?”, and “How do you think significant weight loss should be defined?”.

**Results:**

Over 27% of clinicians advocated removing the AAN diagnosis or combining it with Anorexia Nervosa, while nearly 15% reported concerns about the requirement for ‘significant weight loss’. Over 87% of clinicians suggested ways (often inconsistent across clinicians) to define ‘significant weight loss’, with emphasis on the need for a specified rate (i.e., amount of loss/time) and consideration of physical health impacts.

**Conclusion:**

Clinicians broadly agree that revisions are necessary to the current AAN definition. However, while some propose specific modifications (e.g., defining ‘significant weight loss’), others advocate for the complete removal of the diagnosis. The breadth of suggestions for how to define ‘significant weight loss’ highlights the ongoing lack of consensus on AAN's relevance as a diagnostic entity.

**Summary**

- Most clinicians identified a need for a clearer definition of “significant weight loss”, but recommended criteria differed substantially.
- Clinicians want clearer identifiers and qualifiers of Atypical Anorexia Nervosa beyond the weight loss criterion.
- Clinicians had conflicting views on the value of a separate Atypical Anorexia Nervosa diagnosis.

## 1 Introduction

Atypical Anorexia Nervosa (AAN) was introduced into the Diagnostic and Statistical Manual of Mental Disorders (DSM‐5; American Psychiatric Association [APA], 2013) to categorise individuals with significant eating disorder pathology and restriction of intake, but without the resulting low body weight needed for an Anorexia Nervosa (AN) diagnosis. To be diagnosed with AAN, all criteria for AN need to be met (restriction of intake, intense fear of gaining weight or of becoming fat, undue influence of body weight or shape on self‐evaluation) but “despite significant weight loss, the individual's weight remains within or above the normal range” (APA 2013). However, the DSM‐5 lacks precise guidance on what constitutes ‘significant weight loss’, when the loss had to occur, and at what rate. Such an imprecise definition of AAN has led to various operationalisations of ‘significant weight loss’ in research settings, with some researchers overlooking the weight loss criterion altogether (e.g., Beard and Waller 2024; Harrop et al. 2021; Walsh, Hagan, and Lockwood 2023). This ambiguity is likely to result in inconsistent research findings, which ultimately has a clinical impact on patients (e.g., risk of missed diagnoses, inappropriate treatment).

There have been several research efforts to better define AAN, particularly the ‘significant weight loss’ criterion. For example, Sawyer et al. (2016) propose significant weight loss should be more than 10% of pre‐morbid body weight, while the individual does not become significantly underweight (≥ 90% of median body mass index (mBMI) for age and gender). On the other hand, Forney et al. (2017) and Herb Neff et al. (2024) found that even just 5% weight loss is associated with significant eating disorder pathology. Such inconsistencies have implications for aspects such as identification and prevalence rates. For example, Wade and O'Shea (2015) identified an overall prevalence rate of 1.9% for AAN when operationalising significant weight loss as a reduction of 1.3 kg/m^2^ in BMI. However, when removing this weight loss criterion, an additional 4.7% could be identified. Importantly, those individuals without weight loss demonstrated similar levels of impairment to those with weight loss, as well as compared to individuals with full threshold eating disorder diagnoses (Wade & O'Shea 2015). Similarly, evidence shows that those with AAN have equal or higher levels of eating disorder pathology compared to those with AN or other full threshold eating disorders (Sawyer et al. 2016; Walsh, Hagan, and Lockwood 2023). Thus, appropriate identification is imperative to ensure that suitable treatment and level of care are provided in a timely manner (Manwaring et al. 2024).

Despite a growing body of research into AAN, there remains ambiguity in terms of how eating disorder clinicians perceive the utility of the AAN diagnosis in clinical settings. A key issue in facilitating uptake by clinicians of any such diagnostic criteria is understanding their perspective on what would be important to consider in defining AAN. As clinicians play a pivotal role in the identification of possible AAN, it is important to understand how they use the DSM‐5 diagnosis in practice, and to explore their perspectives on how it could be better defined. Understanding clinicians' perspectives on the AAN diagnosis is crucial, as it can ultimately inform improvements that have a direct impact on patient identification and treatment.

The current study reports on the qualitative element of a larger mixed methods experimental study using an online survey to investigate how clinicians diagnose AAN (Beard, Wade, and Waller 2024). Clinicians were asked to respond to two open‐ended questions: “What changes are required to the DSM‐5 definition of AAN?”, and “How do you think significant weight loss should be defined?”, which were analysed using content analysis. Thus, the aim of the current study is to understand how AAN could be better defined, from clinicians' perspectives.

## 2 Methods

### 2.1 Design

This study is part of a mixed methods experimental study investigating clinician confidence in diagnosing AAN. The quantitative element of the study has been reported elsewhere (Beard, Wade, and Waller 2024). The current study describes the qualitative element, where participants completed an online survey and were asked to answer two open‐ended questions relating to possible refinement of the DSM‐5 AAN definition. The study was approved by the University of Sheffield Ethics Committee (Ref. 051548) and was pre‐registered (https://osf.io/ht53c/). The current study has one deviation from the pre‐registration, as content analysis was used to analyse text responses, rather than thematic analysis. This approach was seen as more appropriate, given the limited number of questions asked of the participants.

### 2.2 Participants

An opportunity sample of 47 clinicians working with eating disorders were recruited through professional contacts and through advertisements to professional organisations and societies. Potential participants were given a link in the recruitment advertisement to an online information sheet and consent form. Those who chose to take part in the study continued to the online Qualtrics questionnaire, which they completed anonymously.

### 2.3 Measure and Procedure

This study describes the qualitative element of a wider study investigating clinician confidence in diagnosing AAN (Beard, Wade, and Waller 2024). Participants completed an online Qualtrics questionnaire requiring them to read twelve short case vignettes based on fictitious patients who varied in their level of weight loss (0%, 5%, 10%, 15%) and end weight (high, normal, borderline low weight/BMI). Participants were asked to rate how confident that each patient would be diagnosed with AAN, or another diagnosis (e.g., AN, Bulimia Nervosa etc.). Following this quantitative element of the study, participants were asked to read the DSM‐5 definition of AAN, then to answer the following two questions: “*What changes do you think are required to the DSM‐5 Atypical Anorexia Nervosa diagnosis, if any*?” and “*How do you think significant weight loss should be defined?”.*

### 2.4 Data Analysis

This study used descriptive qualitative methodology to examine textual responses to the two open‐ended questions. This method aims to ‘stay close’ to the data by describing the perspective of participants rather than explain or interpret them (Sandelowski 2000). Content analysis, guided by the steps outlined in Elo and Kyngäs (2008), was utilised to identify patterns, themes and frequencies within the data. We did not have specific hypotheses about what the data would demonstrate about clinicians' reasoning and opinions about the changes needed to DSM‐5 or the level of weight change that would be significant. Therefore, rather than a deductive approach, an inductive approach was used to explore the data with no preconceived themes or categories in order to discover any unexpected insights. Responses were copied from Qualtrics into a Word document, and one author (JB) became familiar with the responses. [JB] initially created open codes, which formed the basis of the coding sheets. Both authors (JB and GW) then independently used the coding sheet to code the responses to each question. Any discrepancies between the two authors were discussed in order to reach an agreement. Codes were grouped under main categories, generic categories, and subcategories. The authors who coded the responses include a research assistant (JB) who delivers brief cognitive‐behavioural therapy for non‐underweight eating disorders (including AAN), and an experienced eating disorder researcher and clinical supervisor who co‐developed CBT‐T for non‐underweight eating disorders (GW).

#### 2.4.1 Inter‐Rater Agreement on Coding for Content Analysis

Each author independently analysed the data. Overall percentage agreement was high. Agreement on codes within the “*changes suggested to the DSM criteria”* category was 76.3% (Cohen's kappa = 0.749), indicating substantial agreement. Similarly, agreement on codes within the “*how ‘significant weight loss’ should be defined”* category was 85.3% (Cohen's kappa = 0.841). Disagreements and discrepancies (e.g., where one rater identified a code and the other did not) were resolved through discussion until consensus (100% agreement) was achieved.

## 3 Results

### 3.1 Clinician Demographics

Forty‐seven clinicians took part in the study. Table 1 shows the participants' gender, ethnicity, and profession. Their mean age was 46.6 years (SD = 15.1), and the mean number of years spent treating eating disorders was 16.2 (SD = 14.6). Most clinicians worked in a public healthcare setting (46.8%), with the remainder working in private healthcare (21.3%) or a mix of both (31.9%). Whilst the study information sheet did not specify that participants should be trained formally or licenced to diagnose eating disorders, it did outline that participants would be asked to rate how likely they would be to give the patient in each vignette a particular eating disorder diagnosis. All 47 clinicians responded to both questions.

**TABLE 1:** Participant demographic characteristics (*N* = 47).

Characteristic	*N* (%)
Gender	
Male	9 (19.1)
Female	38 (80.9)
Ethnicity	
Any White background	44 (93.6)
Any Asian background	1 (2.1)
Any Hispanic background	1 (2.1)
Prefer not to say	1 (2.1)
Profession	
Clinical psychologist	25 (53.2)
Psychiatrist	6 (12.8)
Eating disorder therapist	6 (12.8)
Medical doctor	3 (6.4)
Nurse/mental health nurse	3 (6.4)
Social worker	2 (4.3)
Other	2 (4.3)

*Note:* Clinical psychologists have a doctoral level degree, whereas eating disorder therapists do not, but they still deliver therapy (e.g., post‐graduate CBT trained).

### 3.2 Question 1: Changes Required to the DSM‐5 Atypical Anorexia Nervosa Diagnosis

As shown in Table 2, six generic categories emerged under the main category of changes required to the DSM‐5 AAN diagnosis: (1) Changes suggested to the weight criterion; (2) Clearer psychosocial and physical identifiers needed; (3) Relationship to other diagnoses; (4) Issues with referring to the Anorexia Nervosa criteria; (5) Remove the diagnosis; (6) No changes needed. Generic categories reached saturation by participant six. Twenty‐two subcategories were identified within the generic categories, and no new subcategories could be identified after participant 43. The generic categories shown in Table 2 are addressed in the text below.

**TABLE 2:** Changes suggested by clinicians to the DSM‐5 Atypical Anorexia Nervosa diagnosis.

Main category	Generic categories	Subcategories	*N* (%)
Changes required to the DSM‐5 AAN diagnosis	Changes suggested for the weight criterion	Define significant weight loss	10 (21.3)
		Rate of weight loss	4 (8.5)
		Change to weight suppression/restriction rather than weight loss	3 (6.4)
		Clarify patient could be at a high weight	3 (6.4)
		Remove arbitrary weight loss criteria	2 (4.3)
		Use of growth curves/course of loss	2 (4.3)
		Duration of problem	1 (2.1)
	Clearer psychosocial and physical identifiers needed	Behaviours and cognitions	7 (14.9)
		Impact/impairment	4 (8.5)
		Risk/malnutrition/medical marker	2 (4.3)
		Clarify whether amenorrhea should be considered	1 (2.1)
		How to use with paediatrics	1 (2.1)
	Relationship to other diagnoses	How to differentiate from no eating disorder	5 (10.6)
		How to differentiate from other eating disorders	4 (8.5)
	Issues with referring to the anorexia nervosa criteria^a^	Conflicts with AN criteria (i.e., reference to “significantly low body weight)”; “even though underweight”; or “denial of seriousness of the current low weight”	7 (14.9)
		Operationalise ‘low weight’	2 (4.3)
	Remove the diagnosis	Combine AN and AAN	7 (14.9)
		Remove the term ‘atypical’	3 (6.4)
		Remove the AAN diagnosis	3 (6.4)
	No changes needed	None	5 (10.6)
		Unsure	2 (4.3)
		Unclear	2 (4.3)

*Note:* Percentages do not total 100% as some clinicians' responses included more than one code.

^a^ Where the DSM‐5 AAN definition states “all of the criteria for anorexia nervosa are met, except that despite significant weight loss, the individual's weight is within or above the normal range” (APA 2013, 353).

#### 3.2.1 Changes Suggested for the Weight Criterion

Table 2 shows that over half of the clinicians (*N* = 25, 53.2%) highlighted the need for clearer guidance on the weight criterion for AAN. The subcategory with the highest count was the need for a specific definition of ‘significant weight loss’ (*N* = 10, 21.3%). The responses to the second survey question aim to understand how clinicians think this definition should be established (see Table 3). Similarly, clinicians emphasised the need for a specified rate of weight loss (amount/time), and to consider the duration of the problem. Others stated that guidance should be provided on using patients' growth curves and consideration of the course of the weight loss.

**TABLE 3:** Clinician opinions on how “significant weight loss” should be defined.

Main category	Generic category	Subcategories	*N* (%)
How “significant weight loss” should be defined	Rate of loss	Needs to be defined as a specific rate (i.e., a specific amount over a specific time frame)	13 (27.7)
		Using criteria for malnutrition	1 (2.1)
		> 2 lbs per week consistently over 6 weeks	1 (2.1)
		Rate of loss should be quick/weight loss should be over a short period of time	4 (8.5)
	Percentage loss	≥ 10% of body weight	6 (12.7
		As a percentage of body weight	6 (12.7)
		5% body weight plus body image concerns	1 (2.1)
		10% body weight for youth, 15% body weight for adult	1 (2.1)
		20% body weight	1 (2.1)
		As a percentage and/or set weight	3 (6.4)
	Level of restriction	Severe restriction/abnormal eating/out of balance methods to lose weight	2 (4.3)
		Lack of insight that dietary behaviours are unhealthy	1 (2.1)
	Individualised criteria	Allow for clinical judgement	1 (2.1)
		Age dependent	1 (2.1)
		Restricted diet may not lead to weight loss	1 (2.1)
		Weight history	1 (2.1)
		Dependent on start weight	1 (2.1)
		Specified range	1 (2.1)
		Weight loss trajectory	1 (2.1)
		Growth curves	2 (4.3)
		Should not be based on BMI/should not be arbitrary	4 (8.5)
	Health related qualifiers	Menstrual function	2 (4.3)
		Medical instability/impact on physical health/malnutrition	11 (23.4)
		Cognitive impairment/impact on mental health	2 (4.3)
		Impairment on everyday functioning	1 (2.1)
	No changes to definition	No change needed to the definition	1 (2.1)
		Unsure	4 (8.5)
		Unclear	1 (2.1)

*Note:* One clinician stated changes are required to all atypical eating disorders. Percentages do not total 100% as some clinicians' responses included more than one code.

In contrast, some clinicians believe the weight loss criterion should be changed to ‘weight suppression’ or ‘restriction’, but only two clinicians suggested the arbitrary weight loss criterion should be removed altogether. Three clinicians stated that it needs to be made clearer that AAN patients can be at a high weight. The DSM‐5 does state “the individual's weight remains within or above the normal range”, so it is possible these clinicians are referring to the AN criteria that are referenced in the AAN definition (discussed below).

#### 3.2.2 Clearer Psychosocial and Physical Identifiers Needed

Around a third of clinicians stated the need for clearer identifiers of AAN beyond just ‘significant weight loss’. The most frequent suggestion was to include more information on eating disorder behaviours and cognitions (e.g., level of restriction, presence of bingeing and/or purging, fear of weight gain). Clinicians also emphasised the importance of considering the level of impact or impairment on areas such as relationships and quality of life. Additionally, some clinicians highlighted the need for an indicator of malnutrition or medical risk, and similarly, whether amenorrhea should be considered. One clinician also stated the need for guidance on how to apply the diagnosis to paediatric populations.

#### 3.2.3 Relationship to Other Diagnoses

About a fifth of clinicians highlighted challenges with differentiating AAN from other diagnoses, or indeed, no eating disorder diagnosis at all. Five clinicians expressed concern about potentially misdiagnosing those without an eating disorder with AAN, due to a lack of guidance on how to differentiate the two. For example, one clinician stated: “*I'm concerned that every individual living in a larger body and trying to lose weight will be diagnosed with AAN*”. Similarly, four clinicians stated the difficulty of differentiating AAN from other eating disorders. For example, one clinician stated: “*Better clarification around what significant weight loss is (as people lean towards Bulimia diagnosis if binge eating is present, despite weight loss)*”. These concerns echo the above call for clearer psychosocial and physical identifiers.

#### 3.2.4 Issues With Referring to the Anorexia Nervosa Criteria

A fifth (19%) of clinicians identified issues with referencing the AN criteria in the AAN definition (“All of the criteria for anorexia nervosa are met, except that…“; APA, 2013, 353). The DSM‐5 criteria for AN makes several references to ‘low body weight’ (e.g., “Restriction of energy intake relative to requirements, leading to a significantly low body weight…“), which clinicians highlighted as being incompatible with the main differentiating factor between AAN and AN, which is being at a normal or above normal weight for AAN. Some clinicians proposed alternatives to these AN criteria—for example: “*[Restriction of energy intake relative to requirements], leading to a drop in weight*”; “*persistent behaviour that interfering with weight gain regardless of current weight status*”; and “*denial of the seriousness of the weight loss and impact of restrictive behaviours*”.

#### 3.2.5 Remove the Diagnosis

Conflicting with the above four generic categories, 13 clinicians (27.7%) expressed concerns about the value of having a separate AAN diagnosis, questioning whether the diagnosis should be removed in some way, rather than simply amending or clarifying the definition. Interestingly, though some clinicians believe there are issues with referencing the AN criteria in the AAN diagnosis (see above), seven clinicians suggested that AAN and AN should be combined as one diagnosis, emphasising that a weight‐based distinction is stigmatising and leads to weight bias. Three clinicians stated that the term ‘atypical’ should be removed, and three stated that the AAN diagnosis as a whole should be removed. Whilst it was not clear whether these clinicians were also advocating for merging the AAN and AN diagnoses, it is clear that these clinicians also share concerns that the atypical diagnosis could lead to weight bias and stigma, and question its value as a diagnostic entity.

#### 3.2.6 No Changes Needed

Directly opposing the clinicians' statements categorised under the above generic categories, five clinicians (10.6%) stated that no changes are needed to the current DSM‐5 definition of AAN. Although this is a relatively small proportion of clinicians, it demonstrates the very divergent opinions that clinicians hold about how AAN should be defined.

### 3.3 Question 2: How “Significant Weight Loss” Should Be Defined

Table 3 shows the six generic categories identified under the main category of how significant weight loss should be defined: (1) Rate of loss; (2) Percentage; (3) Level of restriction; (4) Individual differences; (5) Health parameters; (6) No changes needed. Generic categories reached saturation by participant 18. Twenty‐eight subcategories were identified within the generic categories, and no new subcategories were identified after participant 45.

#### 3.3.1 Rate of Loss

Table 3 shows that over a quarter (27.7%) of clinicians agreed that the DSM needs to provide a specific rate of weight loss (i.e., amount/time), but did not provide suggestions for what that rate should be. Four clinicians agreed that the rate of weight loss should be quick or over a short period, but again, no clear definition of this rate was suggested. One clinician suggested using criteria for malnutrition, and one specified a loss of at least 2lbs per week consistently over 6 weeks.

#### 3.3.2 Percentage Loss

Eighteen clinicians (38.3%) stated that ‘significant weight loss’ should be defined as a percentage (e.g., 5%, 10%, 15%) of start weight. Nine clinicians provided specific suggestions for this. The majority (*N* = 6) agreed on 10% body weight loss, one suggested 20%, one suggested 5% with the addition of body image concerns, and one suggested 10% for youths and 15% for adults. The other nine clinicians did not offer a specific suggestion, but stated the DSM should provide a specific percentage of weight loss, or provide ‘and/or’ criteria whereby the patient should show a certain percentage of weight loss and/or have lost a specific amount of weight (i.e., kg or lbs).

#### 3.3.3 Level of Restriction

Only three clinicians stated that level of restriction should be taken into account when considering whether weight loss has been ‘significant’. It is possible that these clinicians are alluding to the problem identified in the first question, which states there is currently no guidance on how to distinguish someone with AAN versus someone without an eating disorder who might be dieting to lose weight. These clinicians highlighted the need to specify that the restriction is extreme or out of balance, for example: “*Losing a significant percentage of body weight in a comparatively short amount of time through severely restrictive eating (< 75%–50% of BMR)*”. However, it is important to note that this suggestion came from only a very limited number of clinicians.

#### 3.3.4 Individualised Criteria

In contrast with the above three generic categories, which propose more specific, universal definitions of ‘significant weight loss’, 12 clinicians (25.5%) believed that the definition needs to be flexible to meet individual differences. However, there was little consistency among clinicians in how this should be done, or what individual differences should be taken into account. Table 3 provides an overview of these categories, which vary from aspects such as age and start weight, to more vague suggestions such as allowing for clinical judgement. The only subcategories with more than one count stated that the individual's growth curves should be considered (*N* = 2, 4.3%), and emphasised that ‘significant weight loss’ should not simply be based on BMI or other arbitrary measures (*N* = 4, 8.5%). Thus, although a quarter of clinicians agreed that there is not a one‐size‐fits‐all approach to defining significant weight loss, the results suggest a lack of consensus among clinicians on what individual differences do need to be taken into consideration.

#### 3.3.5 Health Related Qualifiers

Similar to the ‘individualised criteria’ category, 16 clinicians stated that the ‘significant weight loss’ definition needs to include an indicator of impact on physical and/or mental/cognitive health, which understandably will vary on an individual basis. For example, assessment of whether weight loss has been ‘significant’ can be ascertained by whether there has been a resulting medical instability (e.g., bradycardia, orthostatic blood pressure) or whether menstrual function has been impacted.

#### 3.3.6 No Changes Needed

Only one clinician believed the current DSM‐5 definition of ‘significant weight loss’ is sufficient, stating “*definition is already given in the first criteria (impact on physical health)*”. This clinician is likely referring to the first AN criterion referenced (“Restriction of energy intake relative to requirements leading to a significantly low body weight in the context of age, sex, developmental trajectory, and physical health”.). Four clinicians were unsure how ‘significant weight loss’ should be defined. It could therefore be concluded that clinicians in this study overwhelmingly agreed that the current definition of ‘significant weight loss’ is insufficient, but their suggested alternative approaches did not coalesce into a clear method of defining AAN.

## 4 Discussion

The aim of this study was to investigate eating disorder clinicians' perspectives on refining the DSM‐5 AAN diagnosis, and thus determine whether there are consistent clinician recommendations for future efforts to clarify the diagnosis in future iterations of the DSM. Content analysis was used to analyse 47 clinicians' responses to two open‐ended questions: “*What changes do you think are required to the DSM‐5 Atypical Anorexia Nervosa diagnosis, if any*?” and “*How do you think significant weight loss should be defined?”*.

Clinicians overwhelmingly favoured revising the current DSM‐5 definition of AAN. Only five clinicians (10.6%) stated no change is needed, highlighting a strong consensus that the definition is inadequate. The most frequent concern, echoing previous qualitative research (Dimitropoulos et al. 2019), was how to operationalise ‘significant weight loss’. This suggests that clinicians believe weight loss remains a relevant criterion for diagnosing AAN, and that they wish to have clear guidance on how to define it in the absence of the low body weight criterion typically associated with AN.

Over a quarter of clinicians (27.7%) believed that significant weight loss should be defined clearly as a specific rate (i.e., a specified amount over a specified period). In addition, clinicians reported needing clearer identifiers and qualifiers of AAN beyond just the weight criterion. For example, they were seeking definitions relating to the presence of eating disorder behaviours and cognitions (e.g., bingeing, purging, fear of weight gain) and medical markers of impact (e.g., bradycardia, amenorrhea). This search for clarity is important, as previous research has found that even just 5% weight loss is associated with elevated eating disorder pathology and distress, but only when combined with cognitive concerns (Forney et al. 2017). Notably, Forney et al. (2017) found that this definition effectively distinguishes AAN from weight loss in the absence of cognitive concerns. They highlight that this distinction avoids the over‐pathologising of weight loss more generally, which was a concern raised by clinicians in the current study. It can thus be summarised that clinicians would like clear guidelines on what constitutes ‘significant weight loss’, and the accompanying characteristics that would need to be present in order to differentiate AAN from other eating disorder diagnoses, or from healthy weight loss in general.

On the other hand, 13 clinicians (27.7%) disagree with clarifying or amending the AAN definition, proposing its removal instead. Their primary suggestion was to combine AN and AAN into one restrictive eating disorder diagnosis. This suggestion aligns with evidence that AN and AAN demonstrate similar levels of physical impairment and psychopathology (e.g., Sawyer et al. 2016; Wade & O'Shea 2015), suggesting that these clinicians do not believe a weight‐based distinction (which was supported by a larger number of eating disorder clinicians here) has clinical value. Furthermore, this group suggested that merging the diagnoses could help reduce weight stigma, which has been associated with delayed care, prolonged eating disorder behaviours, suboptimal treatment environments, and weight discrimination (Harrop et al. 2023).

Evidently, there is disagreement among clinicians regarding the value of a weight loss criterion for AAN and of a weight‐based distinction between AN and AAN. Further research is needed to investigate the clinical utility of a distinct AAN diagnosis, considering the possibility of combining AN and AAN into one restrictive eating disorder diagnosis. Nonetheless, the issue of identifying individuals who will fall into such a category will likely remain. To address this, future iterations of the DSM should provide clear guidance on characteristics associated with this group (beyond any weight‐based criterion), regardless of whether a separate or combined diagnosis exists. Previous research has already highlighted characteristics typically associated with AAN, including eating behaviours (e.g., Jablonski et al. 2024), psychopathology (e.g., Davenport et al. 2015; Johnson‐Munguia et al. 2024), and medical complications (e.g., Nagata et al. 2024; Sawyer et al. 2016). Delphi studies could be a valuable approach to achieve consensus on which characteristics need to be considered as key in the DSM. Not only could this approach provide guidance on which criteria an individual needs to meet to reach a diagnosis, but it could also offer clinician‐driven indices of severity that could aid treatment decision‐making in clinical practice.

It is important to consider the limitations of this study. Opportunity sampling was used to recruit clinicians, with recruitment primarily being advertised in professional organisations. This sample therefore might not be representative of the broader clinician population who diagnose eating disorders. Furthermore, this study focused only on clinicians' perspectives of the AAN definition. Further qualitative research including patient experiences could provide a more complete understanding of the challenges of diagnosing AAN under the current DSM‐5 definition. Future research could also carry out more detailed verbal interviews with participants to allow for more in‐depth questioning of this topic via other qualitative approaches (e.g., thematic analysis based on more diverse questions), as the current study only analysed text‐based responses to two questions. Finally, we acknowledge that only having one author create the initial codes for this study could increase the risk of bias, although there was high inter‐rater agreement between authors when coding the responses.

## 5 Conclusion

To summarise, there is substantial diversity of opinion among clinicians regarding the utility of the DSM‐5 definition of AAN. Many clinicians emphasised the need for a more nuanced and comprehensive approach, extending beyond the current weight‐based distinction between AN and AAN. Further qualitative and quantitative research is necessary to gain a deeper understanding of the perspectives of clinicians, patients, and other key stakeholders. This research could inform future AAN definitions in DSM, ensuring that it accurately reflects the complexities of this condition and enhances clinical understanding and interventions.

## Author Contributions

Both authors conceptualised the paper and contributed to writing the final manuscript and approved the submitted version. Both oversaw the data collection and conducted the data analyses.

## Conflicts of Interest

The authors declare no conflicts of interest.

## Data Availability

The data that support the findings of this study are available from the corresponding author upon reasonable request.
